# Generation of 5-Strand Hamstring Autograft for Anterior Cruciate Ligament Reconstruction—A Technical Note

**DOI:** 10.1016/j.eats.2023.07.006

**Published:** 2023-10-02

**Authors:** Edgar Garcia-Lopez, Ramesh B. Ghanta, Ryan T. Halvorson, Brian Feeley

**Affiliations:** Department of Orthopaedic Surgery, University of California San Francisco, San Francisco, California, U.S.A.

## Abstract

Hamstring tendons are a very commonly used autograft for anterior cruciate ligament (ACL) reconstruction. Given that larger hamstring graft diameter has been shown to positively affect outcomes after ACL reconstruction, several techniques have been developed to optimize this variable. In this technical note, we describe the operative technique for generation of a 5-strand hamstring autograft via tripling of the semitendinosus tendon and doubling of the gracilis tendon, which can serve to maximize graft diameter, especially in patient populations with undersized hamstring tendons at baseline.

Hamstring autografts are frequently used in anterior cruciate ligament reconstruction (ACLR). Numerous technical factors have been theorized to affect outcomes in ACLR, from graft size and length to tunnel positioning.^1^ Of these variables, graft size has been particularly analyzed for its role in predicting postoperative outcomes. Several recent studies have demonstrated that graft size smaller than 8 millimeters (mm) is associated with higher rates of revision and worse patient-reported outcomes.^2^, ^3^, ^4^

Given this established risk factor for ACLR failure, there has been increased interest in the development of techniques that optimize graft diameter. One such technique, which consists of tripling the semitendinosus tendon and doubling the gracilis tendon to create a 5-strand graft, has gained considerable traction in recent years, as it can allow for utilization of the hamstring graft, even in patients whose hamstring tendons are naturally undersized.^5^^,^^6^ Over time, this method has been adapted and improved to optimize both graft fixation and diameter.^7^

In this technical note, we present the technique of generating a 5-strand hamstring tendon graft secured through an Ultrabutton adjustable loop fixation device (Smith and Nephew, Memphis, TN) for use in ACL reconstruction.

## Surgical Technique

**Fig 1:**
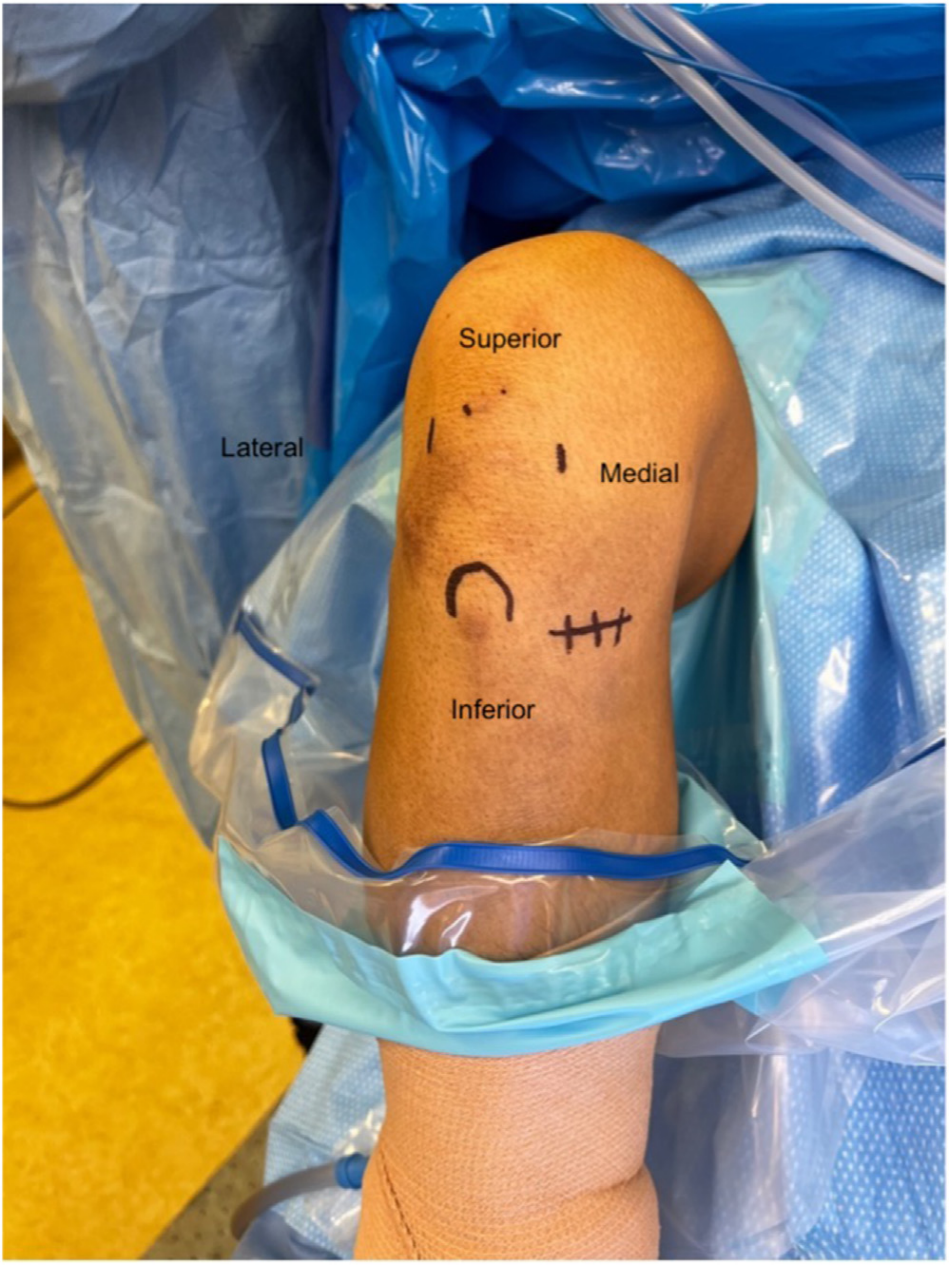
Positioning and setup for anterior cruciate ligament reconstruction of right knee. The patient is placed supine with the right knee flexed to 90° in standard arthroscopic setup. Standard anteromedial and anterolateral portals are marked with an oblique incision made along the medial border of the patella, 2 finger-breadths distal to the joint line extending 2-3 cm for the harvest of hamstring tendons.

After anesthetic induction, an examination under anesthesia is performed, and the patient is placed supine on the operating table with the knee prepared and draped in the usual sterile fashion. Bony landmarks are identified, and standard anteromedial and anterolateral portals are marked. In addition, for hamstring harvest, an oblique incision is marked 2 finger-breaths below the joint line along the medial edge of the patella (Fig 1).

**Fig 2:**
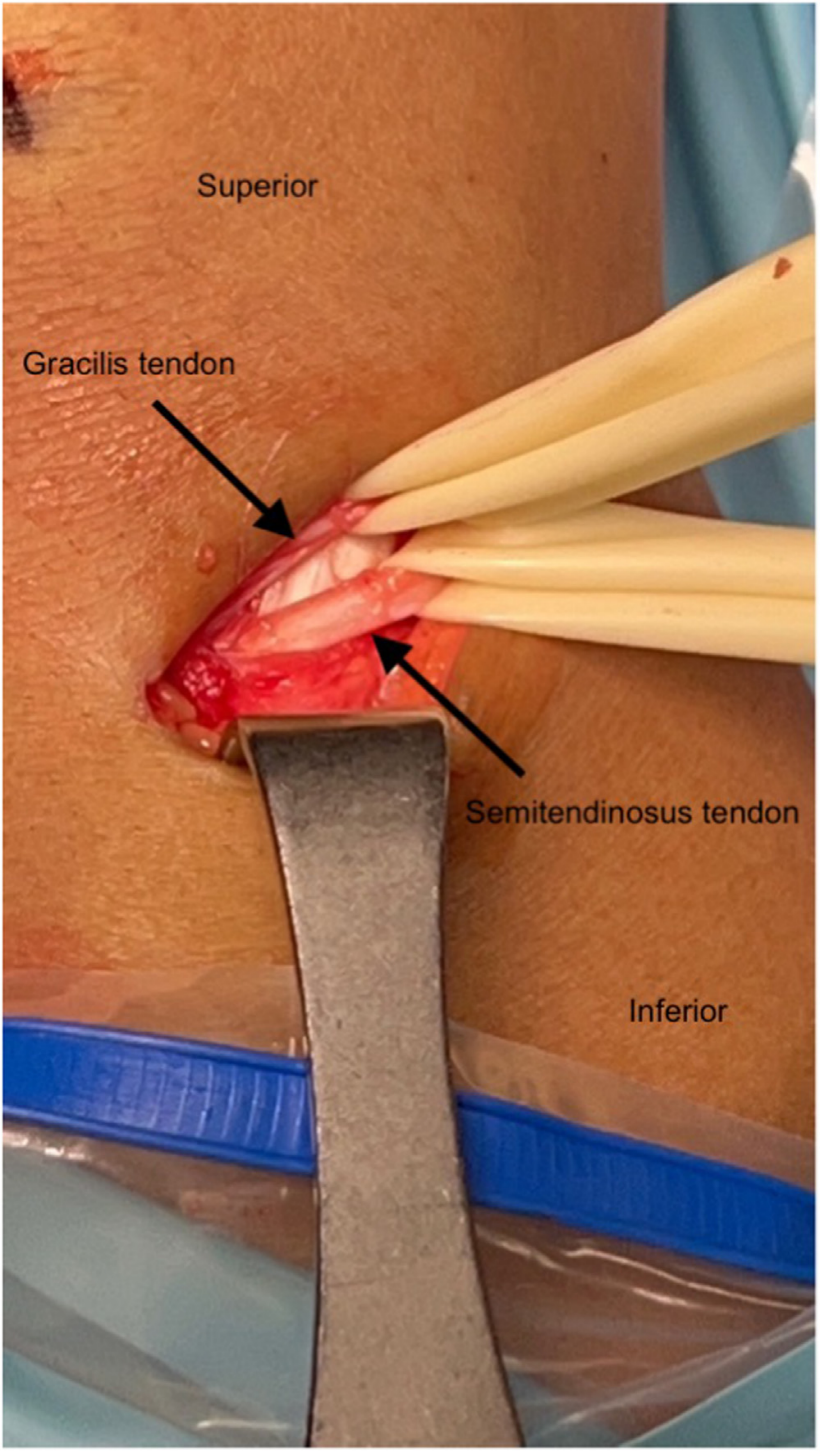
Isolation of hamstring tendons. After sharp dissection to and incision of the sartorial fascia, the gracilis (superior) and semitendinosus (inferior) tendons are identified and isolated via Penrose drains and blunt dissection prior to graft harvesting.

For hamstring harvest, an incision is made with a scalpel, and a sharp dissection is carried down to sartorial fascia. After incising fascia, the semitendinosus and gracilis tendons are identified via palpation. After incision of the superior margins of the tendons, each tendon is isolated with a Penrose drain (Fig 2). A closed stripper is used to harvest both tendons, with caution given, so as not to prematurely cut the tendon. Afterward, each tendon is Krakow stitched with FiberWire (Arthrex, Naples, FL) suture, beginning distally and working proximally, then returning to the distal end.

**Fig 3:**
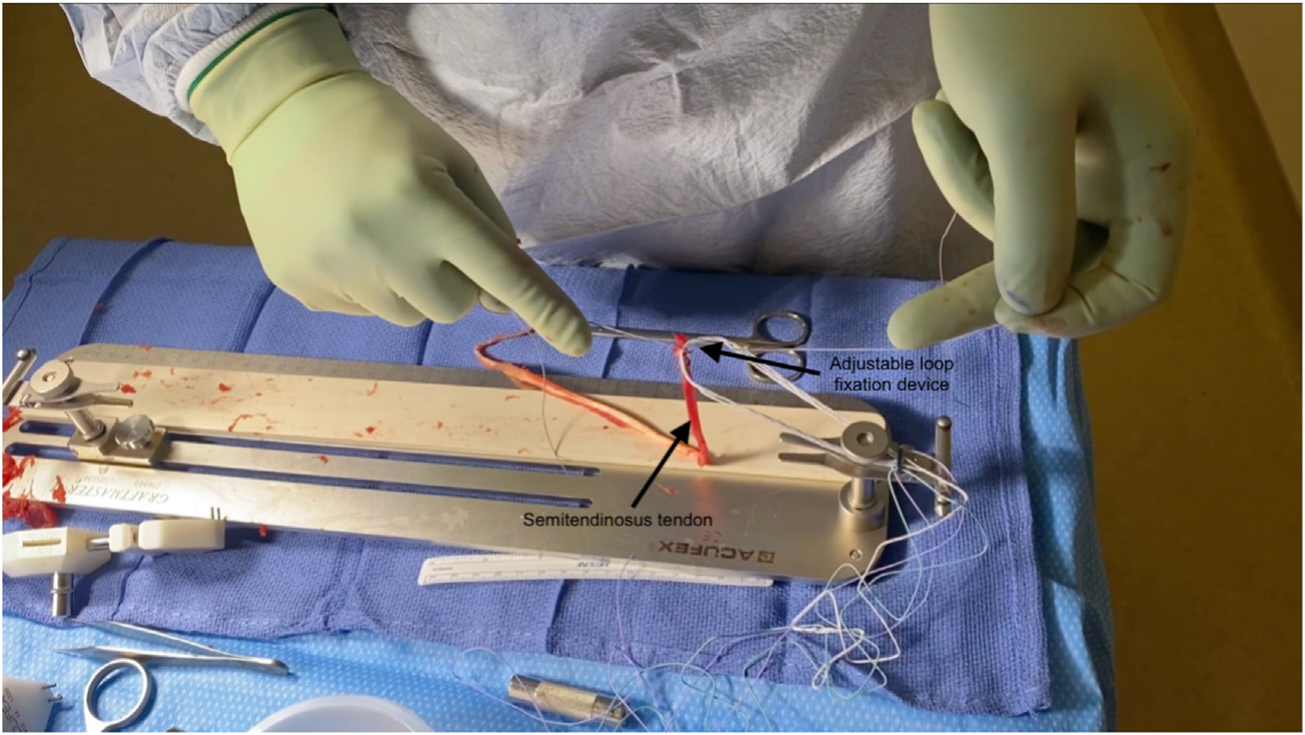
Initial fixation of graft to fixation device. The harvested semitendinosus tendon is taken for the graft preparation site along the sterile back table. After securing the Ultrabutton adjustable loop fixation device (Smith and Nephew, Memphis, TN) to the graft preparation system, the distal end of the tendon is secured to an adjustable loop fixation device through 4 square knots with FiberWire (Arthrex, Naples, FL) suture.

**Fig 4:**
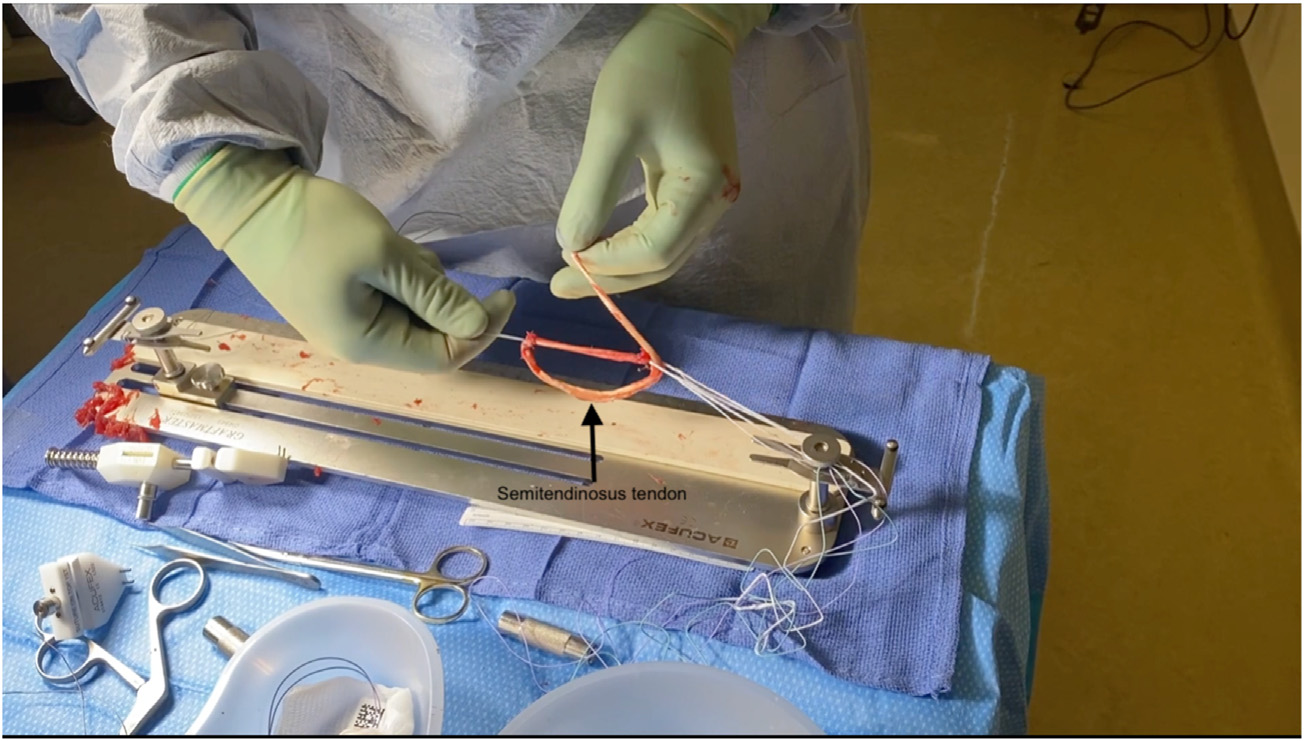
Tripling of the semitendinosus tendon. After the semitendinosus graft is secured to the adjustable loop fixation device at the distal end with FiberWire suture, the free end of the tendon is passed through the suspensory fixation loop of the adjustable fixation device, tripling the graft. A luggage tag stich is then placed with FiberWire suture on the folded end of the graft to facilitate graft holding by passing 2 free ends through the loop created by the tendon and then passing them through the suture loop.

**Fig 5:**
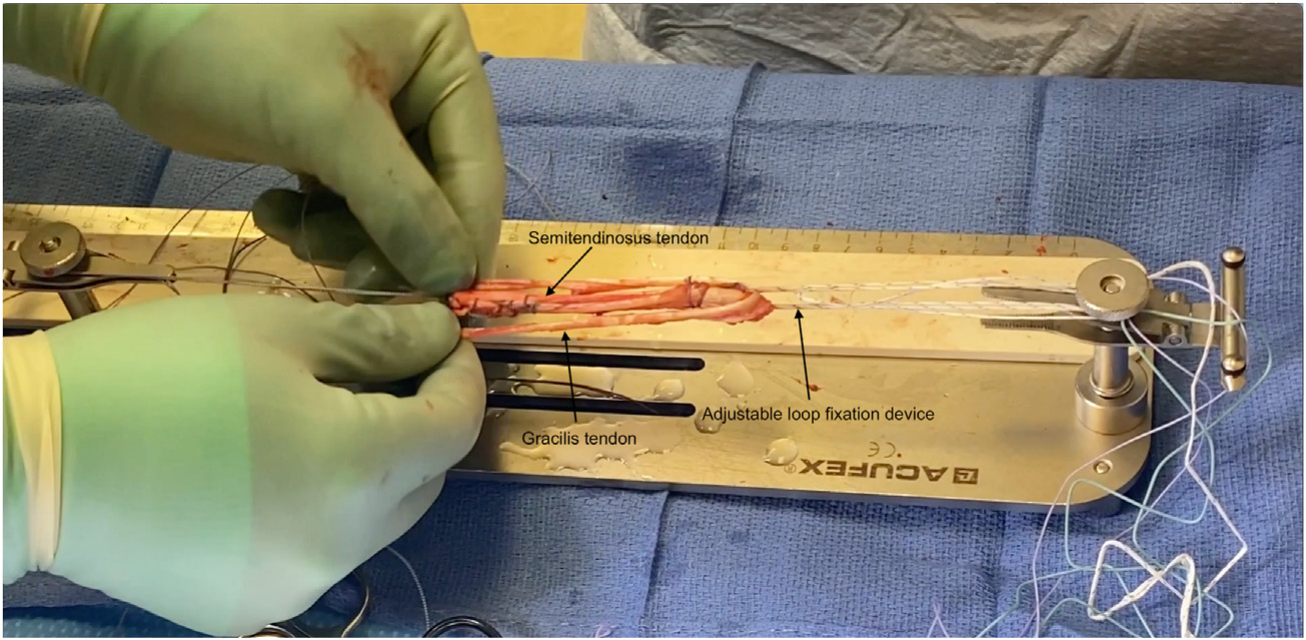
Creation of 5-strand hamstring graft. After the creation and securing of the tripled semitendinosus graft on the graft preparation device, one-half of the harvested gracilis tendon is passed through the suspensory fixation loop of the adjustable fixation device over the tripled semitendinosus tendon, creating a 5-strand hamstring graft.

For graft preparation, the tendon is first sharply debrided of all muscle tissue with scalpel (Video 1). The end of the tendon that was not previously sutured is then secured using a Krakow stitch in a similar manner to that described above. Afterward, the Ultrabutton loop fixation device is attached to the graft preparation system, and the distal end of the semitendinosus tendon is secured to the fixation device via 4 square knots with FiberWire suture (Fig 3). One-third of the free end of the tendon is then passed through the adjustable loop, tripling the graft (Fig 4). A luggage tag stitch is then placed on the folded end to facilitate graft holding. After securing the folded end of the graft to the jig, it is then tubularized with a Krakow stitch through all 3 tendons using FiberWire suture, which is secured using a surgical knot at the end. One-half of the gracilis tendon is then passed through the adjustable loop and laid along the tripled semitendinosus tendon, creating a 5-strand hamstring autograft (Fig 5).

After ensuring that the graft fits snugly through the size 8 cylinder, attention is then given to the knee, where a standard diagnostic knee scope is performed. Afterward, the ACL footprint is debrided from the trochlear notch with a shaver, taking care to note the origin and insertion of the anatomic ACL. The center of the femoral footprint is marked 6 mm anterior to the posterior cortex of the femur with the knee flexed to 90°. Utilizing an inside-out technique, the knee is hyper-flexed to 120°, a guide pin is placed through the eventual femoral tunnel site and driven out the lateral leg, and then over-reamed to create the femoral tunnel. The tibial tunnel drill guide is then placed through the anteromedial portal at the posterior aspect of the anterior horn of the lateral meniscus and the tibial tunnel is drilled arthroscopically. Although viewing from the medial portal, the fixation device sutures holding the graft are first passed through the tibial tunnel and then the femoral tunnel. Then the graft is pulled through using leading suture strands. Once the suture button is passed through the femur, the button-flipping sutures are pulled to ensure the button has flipped and sits flush with the femur. Afterward, the white suture is pulled to reduce and tension the graft following by the placement of the intra-fix implant to secure the graft on the tibial side.

## Discussion

Hamstring graft size diameter has become an increasing point of emphasis in ensuring positive ACLR outcomes in recent years. In a prospective study of 257 patients who had undergone ACLR, Magnussen et al. founded that revision was performed in 1.7% of patients with graft greater than 8 mm in diameter, compared to 6.5% in those with 7.5-8-mm grafts, and 13.6% in grafts less than 7 mm^3^. In the same study, the authors found that the mean graft size for all patients was 7.9 mm, with female patients having a mean diameter of 7.7 mm. Therefore, the data illustrate that undersized hamstring autografts are a relatively common occurrence and more likely to lead to worse outcomes.^3^ To address this, 5-strand hamstring tendon grafts have become popularized, with the technique revitalized after the publishing of the Magnussen study.^5^^,^^6^^,^^8^ Five-strand hamstring grafts have been found to be biomechanically similar with to 4-strand grafts in load to failure and stiffness, while offering a greater diameter.^9^ In recent studies by both Krishna et al. and Wan et al., the authors found that 5-strand hamstring grafts increased the mean graft diameter significantly by an average of 1.4 mm compared to 4-strand grafts.^7^^,^^10^

In this technical note, we present the technique for generating a 5-strand hamstring graft through tripling of the semitendinosus tendon and doubling of the gracilis tendon. Although this technique does increase graft diameter, it sacrifices graft length in order to do so. Although this could theoretically compromise graft incorporation within the tunnels, recent studies have indicated that graft length is not as predictive of outcomes as previously thought (Table 1).^11^, ^12^, ^13^ Therefore, this technique can be performed in patients whose native anatomy would otherwise prevent the utilization of hamstring graft, such as the pediatric population.

**Table 1:** Advantages and Disadvantages of 5-Strand Hamstring Tendon Graft

Advantages	Disadvantages
Increases graft diameter significantly	Decreases graft length, theoretically compromising graft incorporation
Allows for utilization of the hamstring graft in those patients with naturally undersized tendons	Not shown to be clinically superior to 4-strand grafts >8.0 mm

This technique allows for optimization of ACL autograft diameter, which can positively affect postoperative outcomes, as well as expand the patient population in which surgeons can utilize hamstring autografts.

## Supplementary Data

ICMJE author disclosure forms

Video 1 Tripled hamstring tendon autograft consists of a double portion of the tendon with another strand, the central strand, tied to the loop of a suspensory device. This technique is performed to increase the graft diameter. The muscle tissue is first sharply debrided from each tendon. Each graft measured 27 cm in length. Each end of the tendon that was not previously sutured is sutured using FiberWire to Krakow stitch beginning distally to proximally, and then proximally to distally on the opposite side of the tendon. Both tendons were wrapped in half to first estimate size with tubular sizes. In this case, the double-tendon graft easily fit through the size 8 cylinder, so the decision was made to triple the tendon to increase the graft size. The distal end of of the semitendinosus tendon was secured to the adjustable loop fixation device with a surgical knot consisting of 4 square knots. The free ends of the sutures were then cut at the knot. One-third of the tendon was passed through the suspensory loop fixation device, tripling the graft. A luggage tag stitch was placed through the folded end to facilitate graft holding. At this point, we have our tripled graft. We attach the graft to the jig, so we can tubularize the graft. This is done with a Krakow stitch using FiberWire that goes through all 3 tendons and is finished by tying the ends with 4 square knots. Our graft is now tripled. The nontripled gracilis tendon is now passed through and wrapped in half through the suspensory loop fixation device over the completed semitendinosus tendon. With this technique, the graft is routinely 9 mm in size.
